# DCF versus doublet chemotherapy as first-line treatment of advanced squamous anal cell carcinoma: a multicenter propensity score-matching study

**DOI:** 10.1186/s40164-023-00413-2

**Published:** 2023-07-21

**Authors:** Stefano Kim, Véronique Vendrely, Angélique Saint, Thierry André, Pauline Vaflard, Emmanuelle Samalin, Simon Pernot, Oliver Bouché, Mustapha Zubir, Jérôme Desrame, Christelle de la Fouchardière, Denis Smith, François Ghiringhelli, Angélique Vienot, Marion Jacquin, Elodie Klajer, Thierry Nguyen, Éric François, Julien Taieb, Karine Le Malicot, Dewi Vernerey, Aurélia Meurisse, Christophe Borg

**Affiliations:** 1 Clinical Investigational Center, INSERM CIC-1431, Centre Hospitalier Universitaire de Besançon, Besançon, France; 2 INSERM Unit 1098, University of Bourgogne Franche-Comté, Besançon, France; 3 Department of Oncology, Sanatorio Allende, Cordoba, Argentina; 4 Department of Radiation Oncology, Bordeaux University Hospital, Pessac, France; 5 Department of Oncology, Centre Antoine Lacassagne, Nice, France; 6 Sorbonne Université and Hôpital Saint Antoine, Paris, France; 7 Department of Oncology, Institut Curie, Paris, France; 8 Department of Oncology, Institut du Cancer de Montpellier, Montpellier, France; 9 Department of Oncology, Institut Bergonié, Bordeaux, France; 10 Department of Digestive Oncology, Université de Reims Champagne Ardenne, CHU Reims, Reims, France; 11 Department of Oncology, Hôpital Privé des Peupliers, Paris, France; 12 Department of Oncology, Hôpital Privé Jean Mermoz, Lyon, France; 13 Department of Oncology, Centre Léon Bérard, Lyon, France; 14 Department of Oncology, Bordeaux University Hospital, Bordeaux, France; 15 Department of Oncology, Centre Georges-François Leclerc, Dijon, France; 16 Department of Oncology, University Hospital of Besançon, Besançon, France; 17 Cancéropôle Grand-Est, Strasbourg, France; 18 Hôpital Nord Franche Comté, Montbéliard, France; 19 Department of Gastroenterology and Digestive Oncology, Université Paris-Cité, Georges Pompidou European Hospital, SIRIC CARPEM, Paris, France; 20 Fédération Francophone de Cancérologie Digestive (FFCD), EPICAD INSERM LNC-UMR 1231, University of Burgundy and Franche Comté, Dijon, France; 21 Methodology and Quality of Life in Oncology Unit, University Hospital of Besançon, Besançon, France

**Keywords:** Anal carcinoma, Advanced, Metastatic, Chemotherapy, Docetaxel

## Abstract

Triplet DCF (docetaxel, cisplatin and 5-flurouracil) and doublet CP/CF (carboplatin and paclitaxel/cisplatin and 5-fluorouracil) regimens were prospectively evaluated in advanced squamous anal cell carcinoma (SCCA), and validated as standard treatments. Even though the high efficacy and good tolerance of DCF regimen were confirmed in 3 independent prospective trials, doublet CP regimen is still recommended in several guidelines based in its better safety profile with similar efficacy compared to CF regimen. We performed a propensity score-adjusted method with inverse probability of treatment weighted (IPTW) and matched case control (MCC) comparison among patients with metastatic or non-resectable locally advanced recurrent SCCA, treated with chemotherapy as first line regimen. The primary endpoint was the overall survival (OS), and the secondary endpoint was the progression-free survival (PFS). 247 patients were included for analysis. 154 patients received DCF and 93 patients received a doublet regimen. The median OS was 32.3 months with DCF and 18.3 months with doublet regimens (HR 0.53, 95%CI 0.38–0.74; p = 0.0001), and the median PFS was 11.2 months with DCF versus 7.6 months with doublet regimens (HR 0.53, 95%CI 0.39–0.73; p < 0.0001). The hazard ratios by IPTW and MCC analyses were 0.411 (95% CI, 0.324–0.521; p < 0.0001) and 0.406 (95% CI, 0.261–0.632; p < 0.0001) for OS, and 0.466 (95% CI, 0.376–0.576; p < 0.0001) and 0.438 (95% CI, 0.298–0.644; P < 0.0001) for PFS. The triplet DCF regimen provides a high and significant benefit in OS and PFS over doublet regimens, and should be considered as upfront treatment for eligible patients with advanced SCCA.

**Supplementary Information:**

The online version contains supplementary material available at 10.1186/s40164-023-00413-2.

**To the editor.** The advanced squamous cell carcinoma of the anus (SCCA) is a rare entity but its incidence is steadily increasing [1, 2]. For metastatic or non-resectable locally advanced recurrence, two chemotherapy regimens were prospectively validated [3, 4]. First, the triplet DCF regimen has consistently demonstrated a high objective response rate (ORR, ~85%) and complete response rate (CRR, ~45%), as well as a long-term PFS (24.5% at 5 years) and OS (44.4% at 5 years) rates in three independent prospective trials [3, 5–7], and became standard [8]. The modified biweekly DCF (mDCF) regimen is preferred to the standard DCF (sDCF) regimen due to its good tolerance (grade 3/4 toxicity rate of 36 to 53% with mDCF vs. 83% with sDCF) [3, 7]. Second, carboplatin and paclitaxel (CP) regimen, despite its similar predefined efficacy (ORR 59% vs. 57%) and toxicity (grade 3/4 toxicity rate 71% vs. 76%) endpoints compared to cisplatin and 5-fluorouracil (CF) regimen, was considered as the preferred regimen in a randomized phase 2 study due to its significantly lower serious adverse events [4]. Thus, while there is no safety argumentation to prefer doublet over DCF regimen, and the efficacy data of DCF is encouraging, no direct comparison is currently available.

We used 3 independent large French SCCA databases. All SCCA patients with metastatic or non-resectable locally advanced recurrence, and treated in first-line with at least one cycle of DCF, or a doublet chemotherapy regimen were included in the analysis. The primary outcome was OS, and the secondary outcome was PFS. In order to limit bias due to potential confounding factors unbalanced between treatment groups we applied a propensity score method, considered as the best available tool to minimize the difference of the characteristics among non-randomized groups [9] (Additional File 1).

247 patients fulfilled the eligibility criteria and were included for analysis. 93 patients received a doublet chemotherapy, and 154 patients received DCF (table S1). The median OS was 32.3months (95%CI, 24.8–61.1) in the DCF arm, and 18.3months (95%CI, 13.6–24.0) in the doublet arm (HR 0.53, 95%CI 0.38–0.74; p = 0.0001) (Figure S1). The median PFS was 11.2months (95%CI, 10.1–13.7) in the DCF arm, and 7.6months (95%CI, 6.0-9.1) in the doublet arm (HR 0.53, 95%CI 0.39–0.73; p < 0.0001) (Figure S2). In the matched population (77 patients in each arm) with well-balanced characteristics at baseline (Table S2), the median OS was 61.1months (95%CI, 27.4-NE) in the DCF arm compared to 17.9months (95%CI, 12.1–24.0) in the doublet arm (Fig. 1). The median PFS was 13.1months in the DCF arm (95% CI, 10.6–24.0) versus 7.6months (95%CI, 5.9–9.1) in the doublet arm (Fig. 2). HR for OS and PFS were 0.406 (95%CI, 0.261–0.632; p < 0.0001) and 0.438 (95% CI, 0.298–0.644; p < 0.0001), respectively. In the IPTW analysis, the HR for OS and PFS were 0.411 (95%CI, 0.324–0.521; P < 0.0001) and 0.466 (95%CI, 0.376–0.576; p < 0.0001), respectively. The benefit of DCF regimen was observed irrespectively of doublet chemotherapy regimen used. The HR for OS was 2.34 (95%CI, 1.46–3.73) with CF, 3.07 (95%CI, 1.06–8.84) with CP, and 2.88 (95%CI, 1.41–5.90) with mitomycin and fluoropyrimidine (MF) compared to DCF regimen (Figure S3).

**Fig. 1:**
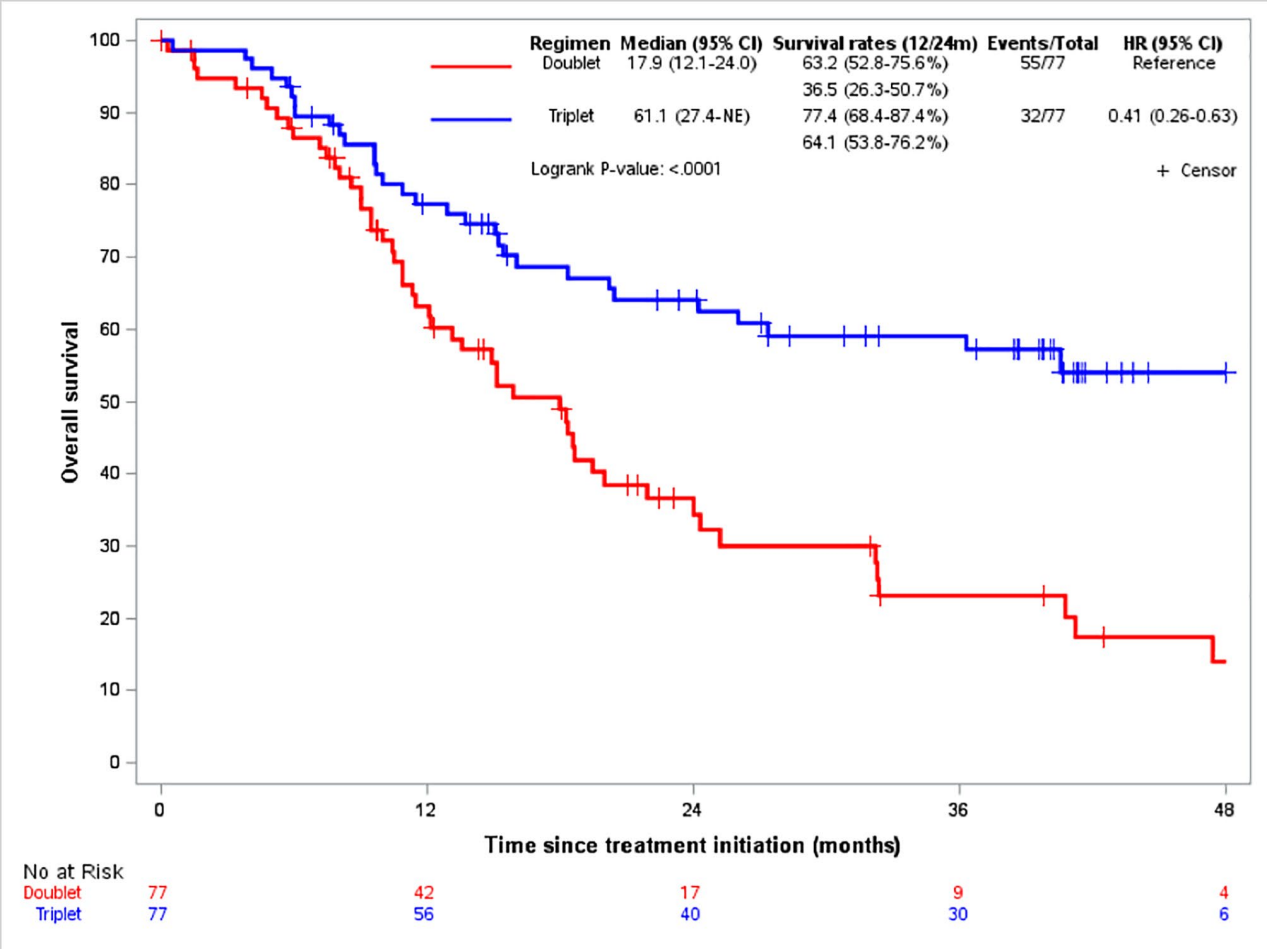
overall survival according to regimens in matched population

**Fig. 2:**
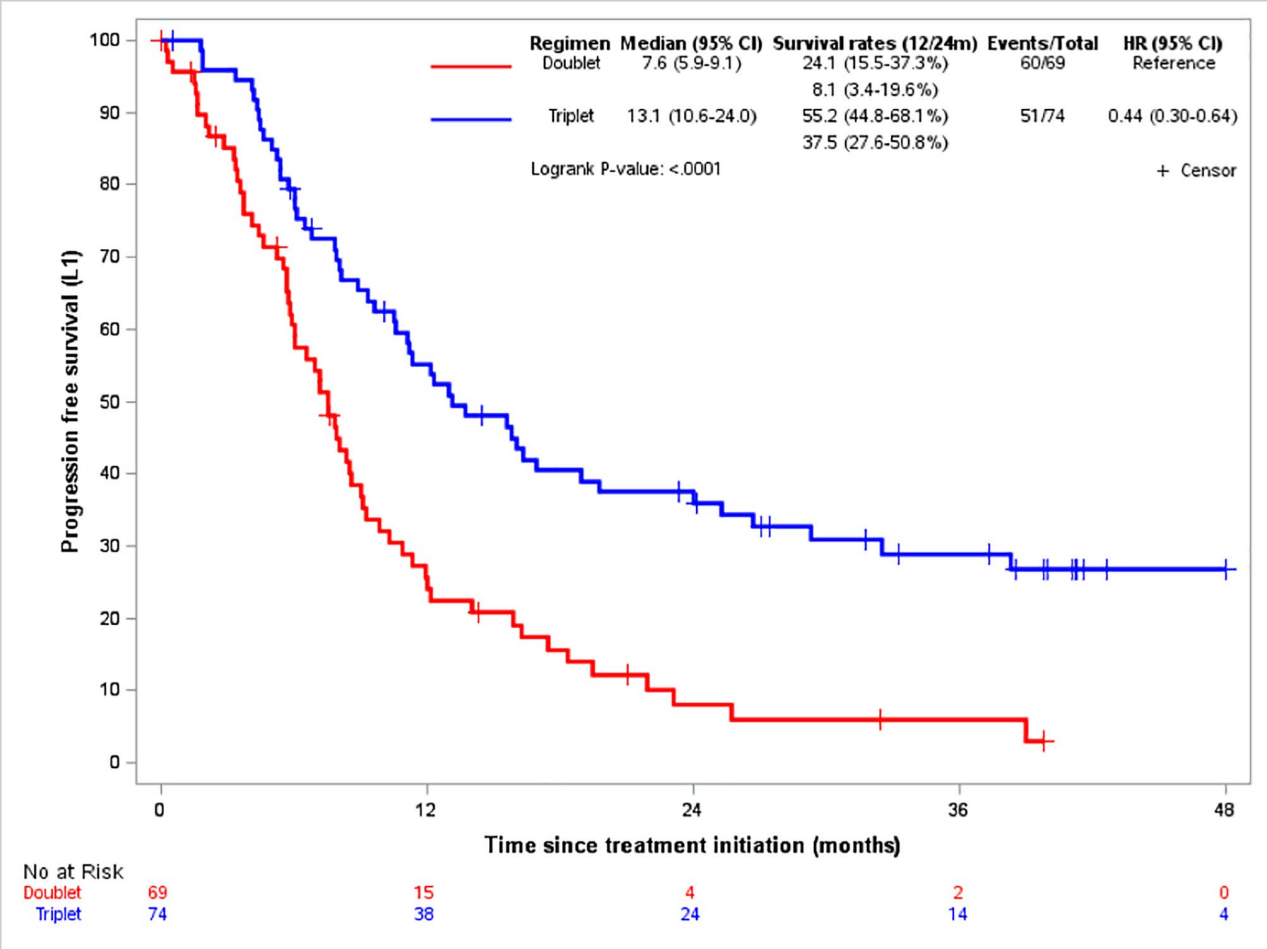
progression-free survival according to regimens in matched population

In this study, the patients’ characteristics and outcomes observed with doublet chemotherapy is comparable to those of published data (Table S3) [4]. Then, DCF regimen provided a high and significant benefit over doublet chemotherapy regimens in the upfront treatment of advanced SCCA patients, irrespective of different doublet regimens. The long-term outcomes also favored DCF: at 4 years, ~55% of patients were alive in the DCF arm, compared to ~15% in the doublet arm. PFS rates were 55.2% vs. 24.1% at 1 year, and 37.5% vs. 8.1% at 2 year, and ~30% vs. <5% at 4 years. These efficacy data are in line with published biological results. In Epitopes-HPV02 and InterAACT trials, the clearance of HPV ctDNA, which was significantly correlated to a better survival, was observed in 61.1% of patients after DCF [10], and 17.9% after doublet CP/CF regimens [4]. Even though there are obvious limitations in our study mainly related to the absence of the randomization and the retrospective nature of the analysis, the magnitude of the adjusted OS benefit was around 60% in favor of DCF. Thus, in the absence of a randomized trial, DCF should be considered as an upfront treatment for eligible patients with advanced SCCA.

In second-line, anti-PD1 immunotherapy is effective in 10–20% of patients. However, new immunotherapy combination regimens currently being evaluated seem more promising. New line of chemotherapy is also an option in patients with good performance status. Besides, ablative treatments should always be considered as part of first and second-line strategies in selective patients, especially in good responders with oligometastatic disease [11].

## Electronic supplementary material

Below is the link to the electronic supplementary material.

Supplementary data: methods, tables and figures

## Abbreviations

CF: cisplatin and 5-fluorouracil
CP: carboplatin and paclitaxel
ctDNA: circulating tumor DNA
DCF: docetaxel, cisplatin and 5-flurouracil
HPV: human papillomavirus
HR: hazard ratio
IPTW: inverse probability of treatment weighted
MCC: matched case control
mDCF: modified DCF
MF: mitomycin and fluoropyrimidine
NE: not evaluable
ORR: objective response rate
OS: overall survival
PFS: progression-free survival
SCCA: squamous anal cell carcinoma
sDCF: standard DCF
