# Supplementary material for: DCF versus doublet chemotherapy as first-line treatment of advanced squamous anal cell carcinoma: a multicenter propensity score-matching study
Source: Exp Hematol Oncol. 2023 Jul 21;12:63. doi: 10.1186/s40164-023-00413-2 (PMC10362607; doi:10.1186/s40164-023-00413-2)
Supplement: Supplementary file 1 — Supplementary data: methods, tables and figures [file 40164_2023_413_MOESM1_ESM.docx]

**Supplementary data**

**Methods**

Patient selection: Three French databases of SCCA patients were used. The first was a national “FFCD-Anabase” database of consecutive patients who started their treatment between January 2015 and April 2020 in 62 French centers, the second was the pooled database from Epitopes-HPV01 and -HPV02 studies from 30 centers, and the third one was a local database from the Lacassagne Cancer Center in Nice of all consecutive patients with advanced SCCA who started their treatment between April 2005 and November 2019. All SCCA patients with metastatic or non-resectable locally advanced recurrence, and treated in first-line with at least one cycle of a triplet (docetaxel, cisplatin, and 5-FU), or a doublet (carboplatin and paclitaxel, cisplatin and 5-FU, mitomycin and 5-FU) chemotherapy regimen were included in the analysis. Exclusion criteria were other than SCC histology, and incomplete data for survival analysis.

Treatment: Patients in the triplet arm received either 6 cycles of standard DCF regimen (sDCF, 75 mg/m^2^ docetaxel and cisplatin on day 1 and 750 mg/m^2^/day of 5-FU for 5 days, every 3 weeks) or 8 cycles of modified DCF regimen (mDCF, 40 mg/m^2^ docetaxel and cisplatin on day 1 and 1200 mg/m^2^/day of 5-FU for 2 days, every 2 weeks). Patients in the doublet arm received either CP regimen (carboplatin AUC 5 on day 1 and paclitaxel, either 175mg/m^2^ on day one every 3 weeks or 80mg/m^2^ on days 1, 8 and 15, every 4 weeks), CF regimen (cisplatin 100mg/m^2^ on day 1 and 5-FU 1000mg/m^2^/day on days 1-5, every 4 weeks; or cisplatin 50mg/m^2^ on day 1 and 5-FU 1200mg/m^2^/day on days 1-2, every 2 weeks), or MF regimen (mitomycin 10-12mg/m^2^ on day 1 and 5-FU 1000mg/m^2^/day on days 1-5, every 4 weeks). Two investigators (AM and SK) have independently checked to identify patients that received triplet and doublet chemotherapy regimens, as well as all inclusion and exclusion criteria.

Outcomes: The primary outcome was the overall survival, defined as the time interval between treatment starting date and death from any cause. Alive patients were censored at the last date known to be alive. The secondary outcome was progression-free survival defined as the time between treatment starting date and the date of the first progression [local, regional, metastatic, or secondary cancer] or date of death from any cause. Patients alive without progression were censored at the last follow-up date.

Statistical analysis: In order to limit bias due to potential confounding factors unbalanced between treatment groups we applied a propensity score method. Propensity score is a statistical method used in non-randomized studies to control confounding factors in a non-parsimonious way. The propensity score was derived from an unconditional multivariate logistic regression which estimates the probability to receive triplet compared to doublet chemotherapy using variables with p-value <0.05 in univariate logistic regression and ECOG performance status due to its importance in overall survival estimation. Performance and adequacy of the model were checked with the area under the curve (AUC) and Hosmer-Lemeshow test, respectively. We first matched one patient with triplet regimen with one patient with doublet regimen based on the propensity score with a caliper of 0.1, hereinafter called “matched sample”. We then applied in a univariate Cox model the inverse of probability of treatment weighting (IPTW) method using the propensity score, hereinafter called “weighted sample”. Additional information is available at supplementary data. Statistical analyses were performed under SAS version 9.4 considering a significance threshold α of 5%.

**Propensity score analysis**

A propensity score, derived from multivariate logistic regression analysis was performed to estimate the probability to have triplet chemotherapy including age <65 years, locally advanced recurrence, and a number of metastatic sites and ECOG performance status (PS table).

The AUC for a multivariate logistic model was equal to 0.7375 and p-value of Hosmer-Lemeshow test was equal to 0.7517, showing a good performance and adequacy of the model (PS figure). The matching algorithm allowed to match 77 patients in each arm. Baseline characteristics of the matched sample are presented in the Table S2, with no statistically significant difference among the parameters involved in the propensity score.

**Table S1: patients’ characteristics of overall population according to regimens**

|  | Overall population  N=247 | Doublet  N=93 | Triplet  N=154 | p-value |
| --- | --- | --- | --- | --- |
| Sex  Male  Female | 66 (26.7%)  181 (73.3%) | 26 (28%)  67 (72%) | 40 (26%)  114 (74%) | 0.7106 |
| Age  Mean (std)  Median (min-max)  Q1-Q3 | 61.3 (10.9)  60.7 (34-86)  52.5-69.3 | 64.2 (12.1)  63.7 (34-86)  55.4-74.8 | 59.5 (9.7)  59.4 (38.6-84)  51.7-66.4 | **0.0026** |
| Age  $\boldsymbol{<}$ 65  $\boldsymbol{\geq}$ 65 | 157 (63.6%)  90 (36.4%) | 50 (53.8%)  43 (46.2%) | 107 (69.5%)  47 (30.5%) | **0.0129** |
| HIV status  Missing  Negative  Positive | 41  188 (91.3%)  18 (8.7%) | 32  56 (91.8%)  5 (8.2%) | 9  132 (91%)  13 (9%) | 0.8584 |
| T  Missing (or x)  is  1  2  3  4 | 51  2 (1%)  10 (5.1%)  58 (29.6%)  65 (33.2%)  61 (31.1%) | 29  0 (0.0%)  2 (3.1%)  18 (28.1%)  23 (35.9%)  21 (32.8%) | 22  2 (1.5%)  8 (6.1%)  40 (30.3%)  42 (31.8%)  40 (30.3%) | 0.8395 |
| N  Missing (or x)  Negative  Positive | 57  43 (22.6%)  147 (77.4%) | 29  13 (20.3%)  51 (79.7%) | 28  30 (23.8%)  96 (76.2%) | 0.7442 |
| Chemoradiotherapy  Missing  No  Yes | 5  75 (31%)  167 (69%) | 0  23 (24.7%)  70 (75.3%) | 5  52 (34.9%)  97 (65.1%) | 0.0962 |
| Surgery of primary tumor  Missing  No  Yes | 6  197 (81.7%)  44 (18.3%) | 0  79 (84.9%)  14 (15.1%) | 6  118 (79.7%)  30 (20.3%) | 0.3075 |
| Stage  Missing  Locally advanced  Synchronous metastases  Metachronous metastases | 6  45 (18.7%)  82 (34%)  114 (47.3%) | 4  10 (11.2%)  38 (42.7%)  41 (46.1%) | 2  35 (23%)  44 (28.9%)  73 (48%) | **0.0254** |
| Number of involved sites  Missing  Mean (std)  Median (min-max)  Q1-Q3 | 7  1.8 (1.2)  1 (1-8)  1-2 | 5  1.5 (0.8)  1 (1-5)  1-2 | 2  2 (1.3)  2 (1-8)  1-3 | **0.0011** |
| Number of involved sites  Missing  1  2  3  >3 | 7  127 (52.9%)  63 (26.3%)  29 (12.1%)  21 (8.8%) | 5  58 (65.9%)  19 (21.6%)  9 (10.2%)  2 (2.3%) | 2  69 (45.4%)  44 (28.9%)  20 (13.2%)  19 (12.5%) | **0.0055** |
| ECOG  Missing  0  1  2 | 1  141 (57.3%)  89 (36.2%)  16 (6.5%) | 1  47 (51.1%)  39 (42.4%)  6 (6.5%) | 0  94 (61%)  50 (32.5%)  10 (6.5%) | 0.2776 |

**Figure S1: overall survival of all patients**


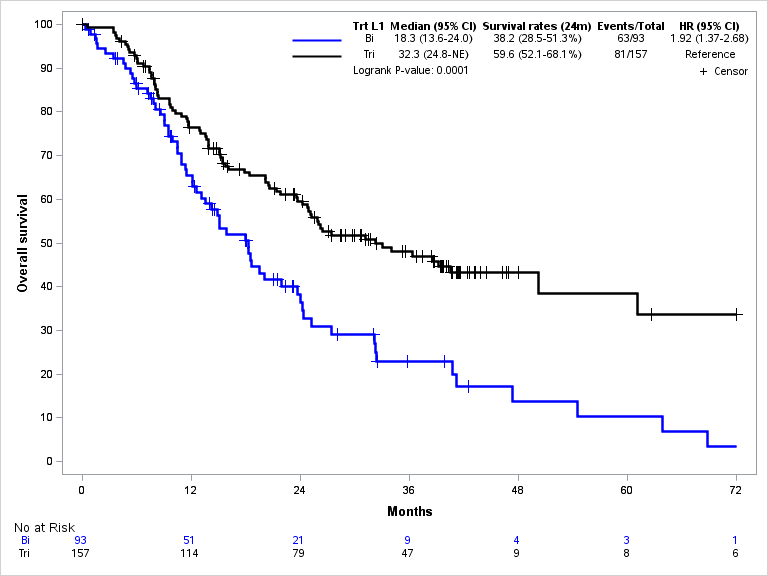


**Figure S2: progression-free survival of all patients**


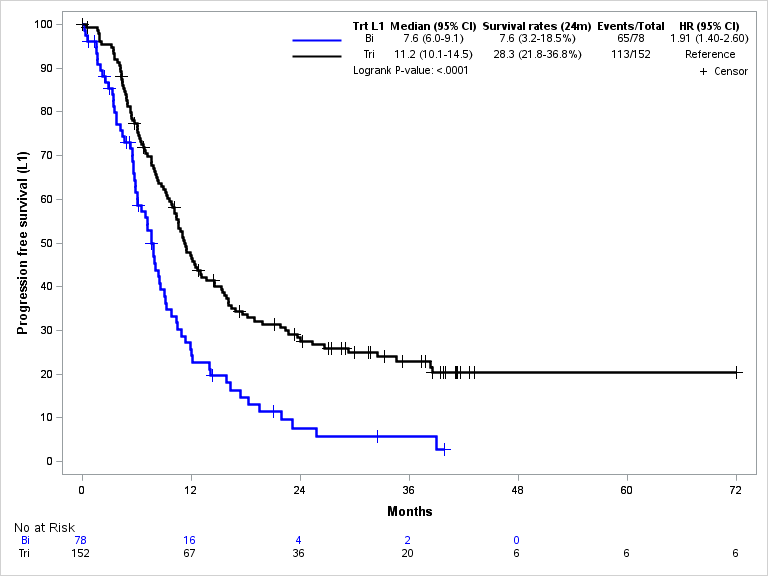


**PS table: Univariate and multivariate unconditional logistic regression to estimate the probability to have triplet compared to doublet chemotherapy in the whole population of analysis**

|  | Univariate analysis | | | | | Multivariate analysis | | |
| --- | --- | --- | --- | --- | --- | --- | --- | --- |
| **Factor** |  |  | OR | 95%CI | P | OR | 95%CI | P |
| **Age** |  |  | 0.960 | 0.936-0.984 | 0.0014 | 0.964 | 0.938-0.990 | **0.0077** |
| **Age** |  |  |  |  |  |  |  |  |
| <65 |  |  | 1 |  |  |  |  |  |
| $\geq$65 |  |  | 0.512 | 0.301-0.870 | **0.0134** |  |  |  |
| **Advanced disease diagnosis** |  |  |  |  |  |  |  |  |
| Locally advanced disease |  |  | 1 |  |  | 1 |  |  |
| Synchronous metastases |  |  | 0.329 | 0.144-0.749 |  | 0.189 | 0.077-0.465 |  |
| Metachronous metastases |  |  | 0.501 | 0.226-1.113 | **0.0274** | 0.363 | 0.155-0.849 | **0.0013** |
| **Number of involved sites** |  |  | 1.598 | 1.191-2.144 | **0.0018** | 2.041 | 1.439-2.896 | **<0.0001** |
| **Number of involved sites** |  |  |  |  |  |  |  |  |
| <3 |  |  | 1 |  |  |  |  |  |
| $\geq$3 |  |  | 2.353 | 1136-4.876 | **0.0213** |  |  |  |
| **ECOG** |  |  |  |  |  |  |  |  |
| 0 |  |  | 1 |  |  | 1 |  |  |
| 1 |  |  | 0.640 | 0.372-1.103 |  | 0.598 | 0.326-1.098 |  |
| 2 |  |  | 0.816 | 0.280-2.380 | 0.2745 | 0.731 | 0.213-2.501 | 0.2493 |

**PS figure: Propensity score, AUC**


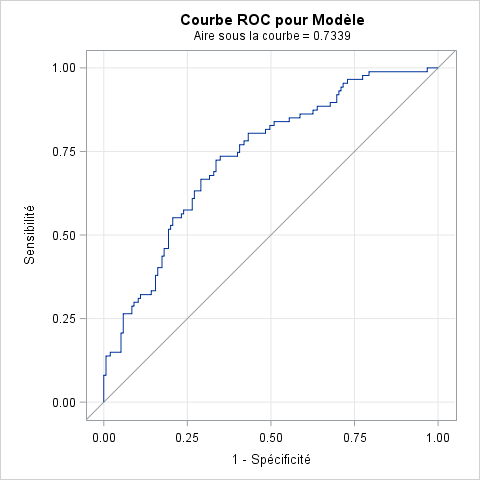


**Table S2: patients’ characteristics for matched population**

|  | Overall population  N=154 | | Doublet  N=77 | Triplet  N=77 | p-value |
| --- | --- | --- | --- | --- | --- |
| Sex  Male  Female | 38 (24.7%)  116 (75.3%) | 22 (28.6%)  55 (71.4%) | | 16 (20.8%)  61 (79.2%) | 0.2621 |
| Age  Mean (std)  Median (min-max)  Q1-Q3 | 62.4 (10.5)  61.8 (34-86)  55.1-69.9 | 62.5 (12.2)  62 (34-86)  52.5-71.8 | | 62.3 (8.6)  60.8 (44.5-84)  57-67.7 | 0.9827 |
| Age  <65  $\boldsymbol{\geq}$65 | 91 (59.1%)  63 (40.9%) | 45 (58.4%)  32 (41.6%) | | 46 (59.7%)  31 (40.3%) | 0.8698 |
| HIV status  Missing  Negative  Positive | 30  114 (91.9%)  10 (8.1%) | 23  49 (90.7%)  5 (9.3%) | | 7  65 (92.9%)  5 (7.1%) | 0.6678 |
| T  Missing (or x)  is  1  2  3  4 | 37  0 (0%)  6 (5.1%)  34 (29.1%)  43 (36.8%)  34 (29.1%) | 25  0 (0%)  2 (3.8%)  13 (25%)  18 (34.6%)  19 (36.5%) | | 12  0 (0%)  4 (6.2%)  21 (32.3%)  25 (38.5%)  15 (23.1%) | 0.4435 |
| N  Missing (or x)  negative  positive | 40  27 (23.7%)  87 (76.3%) | 25  11 (21.2%)  41 (78.9%) | | 15  16 (25.8%)  46 (74.2%) | 0.7976 |
| Chemoradiotherapy  Missing  No  Yes | 0  47 (30.5%)  107 (69.5%) | 0  18 (23.4%)  59 (76.6%) | | 0  29 (37.7%)  48 (62.3%) | 0.0542 |
| Surgery of primary tumor  Missing  No  Yes | 1  127 (83%)  26 (17%) | 0  65 (84.4%)  12 (15.6%) | | 1  62 (81.6%)  14 (18.4%) | 0.6404 |
| Stage  Missing  Locally advanced  Synchronous metastases  Metachronous metastases | 0  20 (13%)  56 (36.4%)  78 (50.6%) | 0  10 (13%)  27 (35.1%)  40 (51.9%) | | 0  10 (13%)  29 (37.7%)  38 (49.4%) | 0.9405 |
| Number of sites involved  Missing  1  2  3  >3 | 0  90 (58.4%)  43 (27.9%)  17 (11%)  4 (2.6%) | 0  48 (62.3%)  19 (24.7%)  9 (11.7%)  1 (1.3%) | | 0  42 (54.5%)  24 (31.2%)  8 (10.4%)  3 (3.9%) | 0.5583 |
| ECOG  Missing  0  1  2 | 0  80 (51.9%)  64 (41.6%)  10 (6.5%) | 0  39 (50.6%)  33 (42.9%)  5 (6.5%) | | 0  41 (53.2%)  31 (40.3%)  5 (6.5%) | 0.9453 |
| Treatment in L1  Missing  CDDP-5FU  Carbo-Taxol  Mitomycine-5FU  DCF | 0  57 (37%)  9 (5.8%)  11 (7.1%)  77 (50%) | 0  57 (74%)  9 (11.7%)  11 (14.3%) | | 0  77 (100%) |  |

**Figure S3: overall survival with different chemotherapy regimens in the matched population**


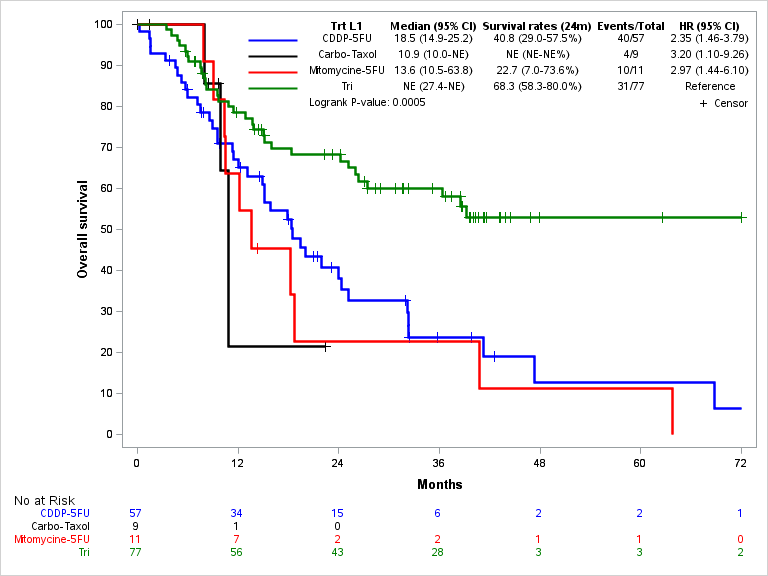


**Table S3**: Patients’ characteristics according to doublet chemotherapies

|  | InterAACT  All population  (n = 91) | | Doublet arm  All population  (n = 93) | | Doublet arm  Matched population  (n = 77) | |
| --- | --- | --- | --- | --- | --- | --- |
| Characteristic | No. | % | No. | % | No. | % |
| Sex  Female  Male | 61  30 | **67**  **33** | 67  26 | **72**  **28** | 56  21 | **73**  **27** |
| Age, years  Median (range)  Mean | **61 (40-75)**  **60** | | **64 (34-86)**  **64** | | **62 (34-86)**  **63** | |
| Extent of disease  Locally advanced  Metastatic | 11  80 | **12**  **88** | 10  79 | **11**  **89** | 10  67 | **13**  **87** |
| ECOG-PS  0-1  2 | 85  6 | **93**  **7** | 86  6 | **91**  **9** | 73  4 | **95**  **5** |
| HIV  Negative/missing  Positive | 86  5 | **95**  **5** | 88  5 | **95**  **5** | 72  5 | **94**  **6** |
| Previous treatment  Radiotherapy | 59 | **65** | 70 | **75** | 59 | **77** |
| No. of metastatic sites  <3  $\geq$3 | 66  25 | **73**  **27** | 77  11 | **87**  **13** | 67  10 | **87**  **13** |
